# Structural Basis of α-Catenin Recognition by EspB from Enterohaemorrhagic *E. coli* Based on Hybrid Strategy Using Low-Resolution Structural and Protein Dissection

**DOI:** 10.1371/journal.pone.0071618

**Published:** 2013-08-14

**Authors:** Mitsuhide Hamaguchi, Hironari Kamikubo, Kayo N. Suzuki, Yoshihisa Hagihara, Itaru Yanagihara, Ikuhiro Sakata, Mikio Kataoka, Daizo Hamada

**Affiliations:** 1 Department of Emergency Critical Care Medicine, School of Medicine, Kinki University, Osakasayama, Osaka, Japan; 2 Research Institute, Osaka Medical Center for Maternal and Child Health, Izumi, Japan; 3 Laboratory of Bioenergetics and Biophysics, Nara Institute of Science and Technology (NAIST), Ikoma, Nara, Japan; 4 National Institute of Advanced Industrial Science and Technology (AIST), Ikeda, Osaka, Japan; 5 Division of Structural Biology, Department of Biochemistry and Molecular Biology, Graduate School of Medicine, Kobe University, Chuo-ku, Kobe, Japan; Universita’ di Padova, Italy

## Abstract

Enterohaemorrhagic *E. coli* (EHEC) induces actin reorganization of host cells by injecting various effectors into host cytosol through type III secretion systems. EspB is the natively partially folded EHEC effector which binds to host α-catenin to promote the actin bundling. However, its structural basis is poorly understood. Here, we characterize the overall structural properties of EspB based on low-resolution structural data in conjunction with protein dissection strategy. EspB showed a unique thermal response involving cold denaturation in the presence of denaturant according to far-UV circular dichroism (CD). Small angle X-ray scattering revealed the formation of a highly extended structure of EspB comparable to the ideal random coil. Various disorder predictions as well as CD spectra of EspB fragments identified the presence of α-helical structures around G41 to Q70. The fragment corresponding to this region indicated the thermal response similar to EspB. Moreover, this fragment showed a high affinity to C-terminal vinculin homology domain of α-catenin. The results clarified the importance of preformed α-helix of EspB for recognition of α-catenin.

## Introduction

**Figure 1:**
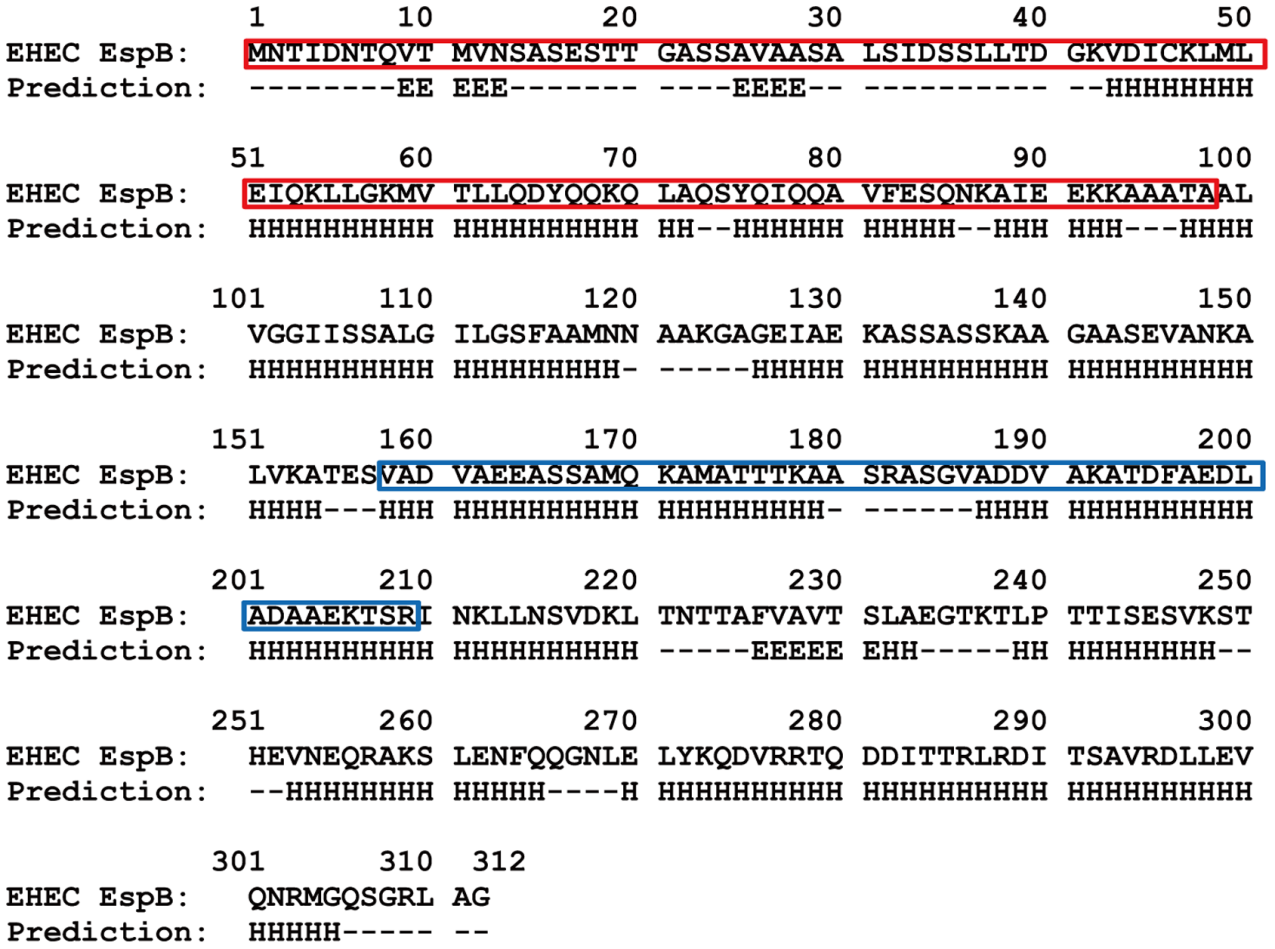
Sequence of EspB from EHEC and the result of secondary structure prediction by Jpred3 (http://www.compbio.dundee.ac.uk/www-jpred/) [49]. The sequence region of α-catenin binding site previously determined [2] is boxed in red and myosin binding sites assigned from the sequence similarity with EPEC EspB [5] is boxed in blue. Regions predicted as helix or strand are indicated as “H” or “E”, respectively.

EspB is one of the virulence factors of enterohemorrhagic *E. coli* (EHEC) [1] that is known to be dependent on a type III secretion system (T3SS). EspB is the multifunctional effector with 312 amino acid residues (Fig. 1) that can bind to various different proteins such as α-catenin [2],[3], α_1_-antitrypsin [4] and myosin [5] from the host cell, and EspA [6] and EspD [7] from the bacterium itself. These interactions of EspB to a range of target proteins are associated with different events in bacterial infection including pore-formation [7], actin reorganization [2],[3],[8] and inhibition of phagocytosis [5].

The binding site for α-catenin within EspB is known to be located at the N-terminal region between residues 1 to 98 (Fig. 1) [2]. However, the precise mechanism of α-catenin recognition by EspB is unclear. Previously, we found that binding of EspB to α-catenin induces dissociation of α-catenin from an E-cadherin/β-catenin/α-catenin triple complex formed at the adherence junction on host cell membrane and enhances the intrinsic ability of α-catenin to promote bundling of actin filaments under *in vitro* conditions [3]. Interestingly, EspB in aqueous solution has the characteristics of a partially folded protein that consists of α-helical secondary structures but only small amount of tertiary contacts if any [9]. This conformational property is similar to that of the molten globule state [10]–[13] which is the compact partially folded state usually accumulated at the early stage of folding kinetics of globular proteins. However, unlike the general molten globule states, EspB does not show an increase of fluorescence intensity of 8-anilinonaphthalene-1-sulfonate [9] which usually binds to surface exposed hydrophobic clusters present in the molten globule intermediates and increase the fluorescence intensity around 480 nm [14]. Moreover, sequence-based disorder predictions as well as a multiplicity of experimental data further suggested that many T3SS-dependent pathogens assume entirely or partially unfolded structures when dissociated from their binding targets [1]. Interestingly, it has been clarified that numerous pathogens from infectious viruses also known to possess a characteristic of intrinsically disordered proteins [15]. Thus, intrinsic disorder could be a generic property for certain classes of virulence factors from infectious bacteria [1] and viruses [15].

Recently, solution NMR has successfully elucidated the structural details of highly disordered proteins. However, such an approach is hardly applicable to EspB due to the line broadening of NMR signals caused by the slow chemical exchange in partially folded regions [9]. Application of another modern high resolution technique such as X-ray crystallography is also impossible because of the difficulty in crystallization of EspB containing significantly flexible regions in the unbound state to its target proteins. We therefore employed a hybrid approach combining low resolution structural and thermodynamic data of intact form and the fragments of EspB obtained by circular dichroism (CD) and small angle X-ray scattering (SAXS) in conjunction with various disorder-order prediction algorithms based on amino acid sequences. The functional properties of the EspB fragments were also tested by fluorescence anisotropy changes upon binding to α-catenin. Our results provide a significant insight into the structural basis of the recognition of α-catenin by EspB.

## Materials and Methods

### Materials

All chemicals used in this study were of analytical grade from Sigma-Aldrich Co. (St. Louis, MO), Nacalai Tesque, Inc. (Kyoto, Japan) or Wako Pure Chemical Industries, Ltd (Osaka, Japan). Synthetic peptides of EspB fragments with an N-terminally attached FITC were purchased from PH Japan (Hiroshima, Japan). EspB was prepared using a combination of Ni^2+^-Sepharose and Resource Q chromatography (GE Healthcare, Milwaukee, WI) as described previously [3],[9]. Recombinant EspB were engineered for expression in *E. coli* BL21(DE3) as N-terminally His_6_-tagged fusion proteins using expression vectors derived from pET28a (Merck Chemicals Ltd., Nottingham, UK). The purified proteins were analyzed without removal of the N-terminal His_6_-tag sequences. α-Catenin635–906 with an N-terminal His-tag was purified using Chelating Sepharose Fast Flow medium (GE Healthcare) charged with nickel chloride in 20 mM sodium phosphate at pH 7.4. After equilibrating the column in 20 mM sodium phosphate at pH 7.4 the protein solution was applied and unbound material was washed away using the same buffer. Bound protein was subsequently eluted using a 0–0.5 M imidazole gradient. The eluted fractions were dialyzed against 20 mM sodium acetate at pH 4.0 and applied onto a SP-Sepharose column (GE Healthcare) equilibrated in 20 mM sodium acetate at pH 4.0, and then eluted using a 0–2.0 M sodium chloride gradient. Fractions containing α-catenin635–906 were pooled and further purified by gel filtration chromatography using a Sephacryl S-200 column (GE Healthcare) equilibrated in 20 mM Tris-HCl (pH 8.0), 0.1 mM EDTA. The protein concentrations of full length EspB and EspB1–176 were determined from UV absorption at 280 nm using an extinction coefficient of 4470 and 2980 M^−1^ cm^−1^, respectively, which were calculated using the ProtParam server (http://www.expasy.org/tools/protparam.html) based on their amino acid sequences. The extinction coefficient for EspB177–312 calculated using the ProtParam server (1490 M^−1^ cm^−1^) was too small to obtain reliable estimations of protein concentration in dilute solution. We therefore employed Bradford’s method [16] to determine the concentration of EspB177–312 using standard curve obtained by full length EspB. The absorption spectra were obtained using a JASCO UV spectrophotometer, V-550 (Jasco Co., Tokyo, Japan).

### CD Spectra

Far-UV CD spectra were monitored using a J-720 spectropolarimeter (Jasco Co.) equipped with a Peltier type thermo-controllable cell holder. A quartz cuvette with a 1 cm pathlength was used for the thermal unfolding experiments and a cell with a 0.1 cm pathlength was used for the analysis of CD spectra of EspB fragments as well as full length EspB. Unless stated otherwise, all data are expressed as mean residue ellipticity. Secondary structure contents of the protein and peptides were analyzed by CDpro [17] using the reference set of SP29.

The thermal unfolding transition was monitored by measuring the ellipticity at 222 nm. The temperature was increased from 10 to 90°C at a heating rate of 1°C min^−1^. The samples cooled to 20°C after the thermal unfolding experiments recovered to the essentially same CD spectra obtained at 20°C before heat treatments, suggesting the unfolding reactions were reversible. The thermal unfolding curves were analyzed by modified method as previously demonstrated [18] assuming the two-state transition between folded and unfolded states based on the following equation,
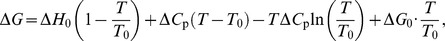
where *T* and *T*_0_ are the given and reference temperatures in Kelvin, Δ*G* and Δ*G*_0_ are the free energy changes upon unfolding of folded state at give temperature *T* and *T*_0_, respectively. Δ*H*_0_ and Δ*C*_p_ is the enthalpy and the heat capacity change upon unfolding reaction, respectively. The ellipticities at 222 nm ([*θ*]_222_) are expressed as 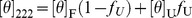, where [*θ*]_F_ and [*θ*]_U_ are the baselines of folded and unfolded species that are linearly dependent on temperature, and *f*_U_ is the fraction of unfolded state. Thus, Δ*G* can be expressed as 
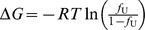
, where *R* is the gas constant. In practice, *T*_0_ was set to 293.15 K ( = 20°C), and all the unfolding curves observed in the presence of different amounts of guanidium hydrochloride (GdnHCl) were simultaneously analyzed by the global-fitting method according to the above equations using the same baselines of [*θ*]_F_ and [*θ*]_U_, Δ*C*_p_ and Δ*H*_0_. IgorPro ver 6.3 (Wavemetrics, Inc., Portland, Oregon)was used for this fitting analysis. The Δ*G*_0_ values were obtained for the individual conditions at different concentrations of GdnHCl. In the absence of GdnHCl, [*θ*]_222_ was linearly decreased as in crease in *T*, and this linear dependence of [*θ*]_222_ was considered as the baseline, [*θ*]_F_. This situation prevented us from obtaining the thermodynamic parameters in the absence of GdnHCl based on the above analysis using the data from the thermal unfolding experiments. We therefore estimated Δ*G* in the absence of GdnHCl (Δ*G*_water_) from the linear dependence of Δ*G*_0_ as a function of the concentration of GdnHCl ([GdnHCl]), i.e. 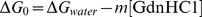, which is generally used for the analysis of denaturant-induced unfolding reaction. The *m* value is the measure of cooperativity of denaturant-induced unfolding reaction which is considered to be correlated with the change in solvent accessible surface area upon unfolding reaction.

### SAXS

SAXS measurements were carried out at a BL-10C synchrotron beamline in the Photon Factory, Tsukuba, Japan. Scattering intensity was monitored by R-axis VII (Rigaku, Tokyo, Japan) and circularly averaged intensity was used for further analysis. A cell with a 1 mm pathlength was used and the protein solution was prepared by dialysis against 10 mM 3-morpholinopropane-1-sulfonate (MOPS) at pH 7.0. The temperature in the cell was maintained at 20°C by circulating temperature-controlled water. Scattering intensities obtained at various concentrations of protein were subtracted from the intensity of the buffer solution without protein using IgorPro ver 6.1 (Wavemetrics, Inc., Portland, Oregon). These values were used to generate Guinier and Kratky plots. *P*_r_ functions were calculated by GNOM [19] using the scattering intensity after reduction of noise by singular value decomposition *via* IgorPro. The *P*_r_ function of phosphotriesterase was calculated using a theoretical scattering curve calculated by CRYSOL [20] based on the atomic coordinates of 1EYW solved by X-ray crystallography [21].

### Fluorescence Anisotropy

Fluorescence spectra were observed using a FP-6500 fluorimeter (Jasco Co., Japan). To observe fluorescence anisotropy, HN32 linear polarizer films (5 cm×5 cm; Polaroid Co., Waltham, MA) were inserted in both excitation and emission lightpaths. The orientations of the polarizers were manually changed to obtain the emission components of *I*_vv_, *I*_vh_, *I*_hv_ and *I*_hh_. *I*_vv_ and *I*_vh_ were the vertical and horizontal emission components obtained for the sample excited with vertically polarized light, and *I*_hv_ and *I*_hh_ were the vertical and horizontal emission components obtained for the sample excited with horizontally polarized light. Anisotropy value, *r* is then defined as,
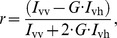
where *G* is defined as *I*_hv_/*I*_hh_. The data were processed by IgorPro ver 6.3 (Wavemetrics, Inc.) to calculate the anisotropy values with different samples and to estimate the dissociation constants, *K*_D_ by nonlinear curve-fitting.

## Results

### Cooperativity of the Thermal Unfolding Transition of EspB

**Figure 2:**
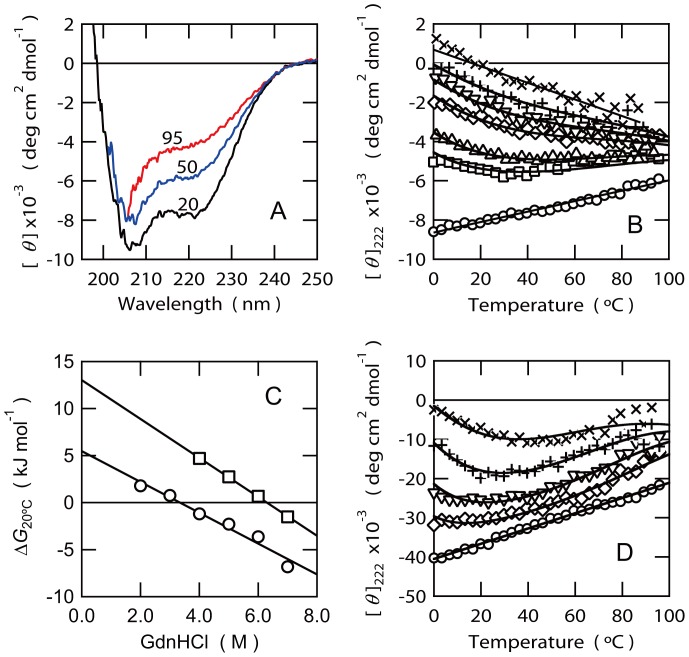
Thermal transition of EspB monitored by CD spectra at pH 7.0 in the presence or absence of GdnHCl. (A) The Far-UV CD spectra of EspB obtained at various temperatures in the absence of GdnHCl. Numbers refer to the temperature in °C. (B) Temperature dependences of the ellipticity at 222 nm ([*θ*]_222_) of EspB in the absence (O) or presence of 2.0 (□), 3.0 (Δ), 4.0 (◊), 5.0 (∇), 6.0 (+) and 7.0 M (X) GdnHCl. Lines are the results of curve-fitting. (C) Linear dependence of Δ*G*_20°C_ against GdnHCl concentrations to estimate the Δ*G*_20°C_ in the absence of GdnHCl (Δ*G*_water_) and *m* value from the slope of the plots. The Δ*G*_20°C_ obtained by the thermal transition curves of EspB and EspB41–70 are shown as circles and squares, respectively. (D) Temperature dependences of the ellipticity at 222 nm ([*θ*]_222_) of EspB41–70 in the absence (O) or presence of 4.0 (◊), 5.0 (∇), 6.0 (+) and 7.0 M (X) GdnHCl. Lines are the results of curve-fitting.

Thermal unfolding transition of EspB at pH 7.0 was monitored by the ellipticity at 222 nm (Fig. 2). Usually, globular proteins with rigid secondary and tertiary structures stabilized by hydrophobic interactions show highly cooperative thermal unfolding transitions represented by a sigmoidal curve that are consistent with a two-state model from the well-ordered and rigid native state to the highly unfolded state. However, EspB displays a noncooperative transition from a α-helical structure to a unfolded state in which the absolute ellipticity at 222 nm decreased almost linearly with increasing temperature (Fig. 2A and circles in Fig. 2B). The lack of cooperativity in the thermal unfolding reaction suggests the lack of highly ordered tertiary contacts stabilized by hydrophobic interactions in EspB. A similar behavior was previously observed for the thermal unfolding of a partially folded or “molten globule” intermediate state of human α-lactalbumin [22]. Thus, the result is consistent with our previous conclusion suggesting that EspB is a natively partially folded protein [1],[9]. Interestingly, further analysis on the response of EspB against the temperature change by far-UV CD revealed the relatively cooperative cold and heat denaturations in the presence of various amount of GdnHCl (Fig. 2B). This result indicates the positive heat capacity change for the unfolding reaction (Δ*C*_p_) of EspB. We analyzed the transition curves assuming two-state transition by global-fitting procedure according to the method described in Materials and Methods. The estimated parameters of unfolding reactions at various GdnHCl concentrations are summarized in Table 1. In this analysis, the estimated Δ*C*_p_ was 0.34±0.01 kJ mol^−1^ K^−1^ or 81.8±1.0 cal mol^−1^ K^−1^. According to the BPpred server (http://www-clarke.ch.cam.ac.uk/BPPred.php) [23], Δ*C*_p_ of a globular protein with 332 amino acid residues, i.e. equivalent length of EspB with hexahistidin-tag, should be 5.15 kcal mol^−1^ K^−1^. The experimentally determined Δ*C*_p_ by thermal unfolding experiment of EspB is therefore extremely smaller than that is expected for the globular protein with the same length of EspB.

**Table 1:** Thermodynamic parameters of unfolding of EspB and EspB41–70 in the presence of GdnHCl at pH 7.0.

GdnHCl	Δ*G*_20°C_	Δ*H*_20°C_	Δ*C*_p_	*m*_GdnHCl_
(M)	(kJ mol^−1^)	(kJ mol^−1^)	(kJ mol^−1^ K^−1^)	(kJ mol^−1^ M^−1^)
EspB				
0.0	5.5±0.7^a^	n/a^b^	n/a^b^	1.6±0.2^a^
2.0	1.8±0.1	−9.1±0.1	0.34±0.01	−b
3.0	0.8±0.1	SAA^c^	SAA^c^	n/a^b^
4.0	−1.2±0.1	SAA^c^	SAA^c^	n/a^b^
5.0	−2.3±0.1	SAA^c^	SAA^c^	n/a^b^
6.0	−3.6±0.1	SAA^c^	SAA^c^	n/a^b^
7.0	−6.8±0.1	SAA^c^	SAA^c^	n/a^b^
EspB41–70				
0.0	13.0±0.2^a^	n/a^b^	n/a^b^	2.1±0.1^a^
4.0	4.7±0.1	−12.6±0.4	0.87±0.02	n/a^b^
5.0	2.7±0.1	SAA^c^	SAA^c^	n/a^b^
6.0	0.7±0.1	SAA^c^	SAA^c^	n/a^b^
7.0	−1.5±0.1	SAA^c^	SAA^c^	n/a^b^

a Estimated from linear extrapolation of Δ*G*_20°C_ obtained by the analysis of the data in the presence of GdnHCl as shown in Fig. 1.

b Not available.

c Same as above.

These results from the CD spectroscopy suggest that either the molecule is in the relatively ordered molten globule-like structure or there is a small relatively structured core and other regions with less-ordered regions in EspB. However, the fact that the EspB does not increase the fluorescence intensity of ANS [9] supports the latter possibility.

### SAXS Indicates the Extended Conformation of EspB

To gain further insight into the conformational properties of EspB, we performed SAXS experiments for this protein at pH 7.0, 20°C. SAXS provides information about the molecular shape and dimension of a macromolecule under a given solution condition. This particular technique is extremely useful when the protein assumes less-ordered structures such as partially folded or highly unfolded states having conformational heterogeneity or ensemble for which more detailed analysis using X-ray crystallography or solution NMR are nearly impossible [24]–[27].

**Figure 3:**
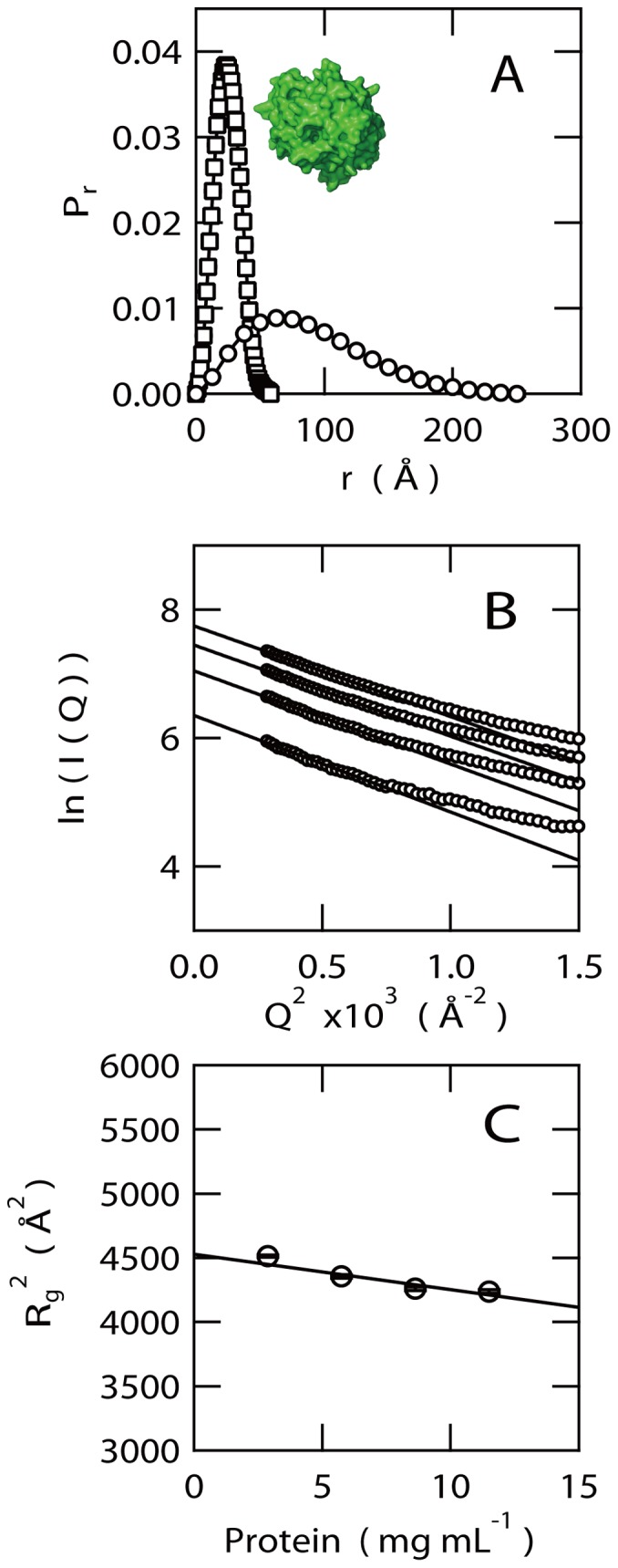
Structural properties of EspB revealed by SAXS. (A) *P*_r_ function of EspB (circles) calculated from SAXS profile and that of phosphotriesterase (squares) calculated from its atomic coordinates (1EYW) downloaded from Protein Data Bank. The structure of phosphotriesterase is represented as a space-filling model (inset) to illustrate the globular nature of the molecule. This model was produced by PyMol (http://www.pymol.org). (B) Guinier plots. Raw data are represented by circles. Lines indicate the results of fitting against linear regions of the raw data. Protein concentrations were 2.0, 5.8, 8.6 and 11.5 mg mL^−1^ from the bottom to the top in panel B, respectively. (C) Protein concentration dependence of square of *R*_g_^2^ calculated from the slope of Guinier plot in panel B.

The pair distance distribution (*P*_r_) function calculated from the scattering intensity of EspB using GNOM program [19] showed an extremely broad distribution with a maximum distance of 250 Å and the optimum position of *P*_r_ at 65 Å (Fig. 3A). For comparison, we calculated *P*_r_ function of phosphotriesterase (364 amino acids) as a typical model of a globular protein whose molecular weight is similar to that of EspB (332 amino acids including hexahistidine-tag) using its atomic coordinates solved by X-ray crystallography (Fig. 3A). Unlike the *P*_r_ function of EspB, the *P*_r_ function of phosphotriesterase indicates an optimum position of *P*_r_ at 24 Å and the maximum distance is ∼60 Å.

We also determined the radius of gyration (*R*_g_) of EspB according to a Guinier plot (ln(*I*(*Q*) vs. *Q* ^2^)) as shown in Figure 3B. The slope of this plot at low *Q* regions corresponds to *R*_g_^2^/3 although the value also depends on the protein concentration. To correct the effect of protein concentration dependency, the *R*_g_^2^ values were plotted as a function of protein concentration and the reduced *R*_g_ value was given by linear extrapolation of this plot to zero protein concentration (Fig. 3C). This analysis provided the *R*_g_ value of EspB of 67.8±0.1 Å, which is slightly larger than the expected *R*_g_ value of urea unfolded state (62.0 Å) according to the empirical equation [28], 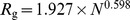, where *N* is the number of amino acids, i.e. 332 in the case of our recombinant EspB with N-terminal hexahistidine-tag.

Scattering intensity obtained by linear extrapolation of the Guinier plot to zero angle, *I*(0) is linearly correlated with the molecular weights of the proteins. Molecular weight of EspB was estimated to be 32,400 by comparison of *I*(0) of lysozyme with a molecular weight of 14,300. This value is close to the calculated molecular weight of monomeric hexahistidine-tagged EspB (34,800) used in this study. Thus, EspB assumes a highly extended monomeric structure comparable to the ideal random coil. Nonetheless, EspB contains folded regions with α-helical secondary structures according to the far-UV CD spectra (Fig. 2) [9].

### α-Helical Structures are Formed at the N-terminal Regions Whereas the C-terminal Half is Highly Unfolded

Although the data from SAXS suggested the highly extended structure of EspB, the protein contains a significant amount of α-helical structures (Fig. 2) [9]. The most α-helical structures may be partially folded according to the noncooperative behavior of thermal unfolding reaction (Fig. 2) Thus, the overall structure of EspB should consist of both relatively ordered and less well-organized α-helical structures. To establish the regions of EspB that adopt α-helical structures, we performed combinatorial analysis using sequence-based disorder-order prediction algorithms and CD spectra of peptide fragments derived from the EspB sequence.

**Figure 4:**
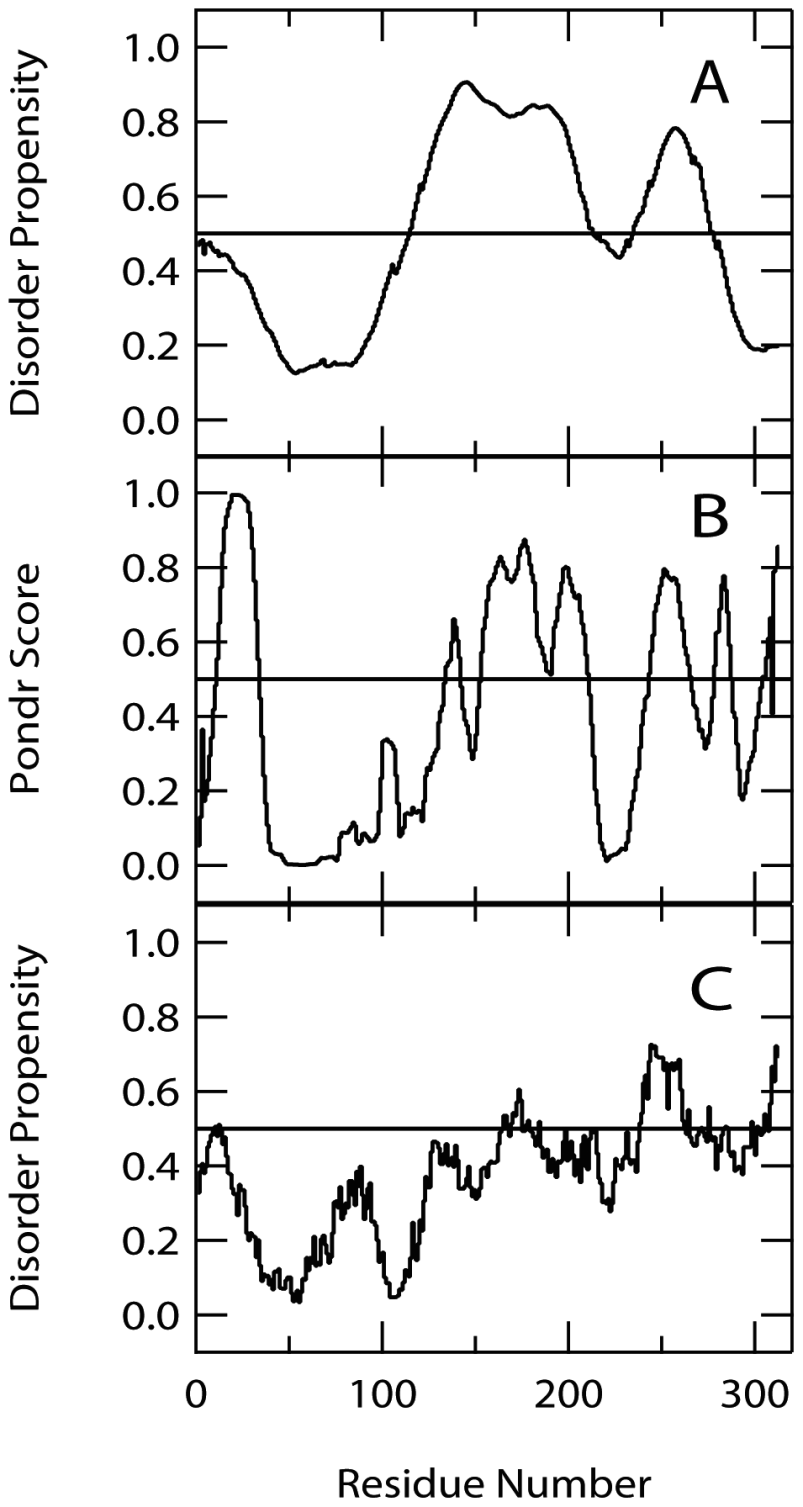
Order-disorder predictions of EspB by using various algorithms. (A) Poodle-L (http://mbs.cbrc.jp/poodle/poodle.html), (B) PONDR (http://www.pondr.com) and (C) IUPred (http://iupred.enzim.hu).

**Figure 5:**
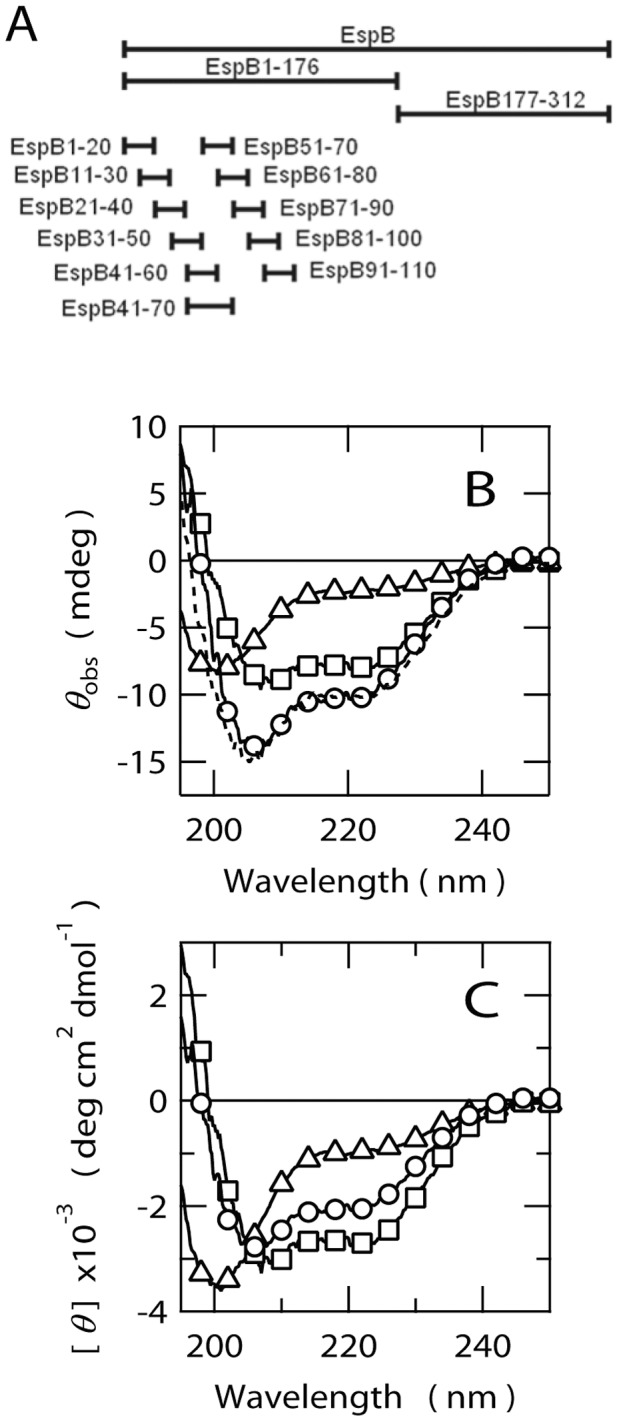
Far-UV CD spectra of EspB fragments. (A) Schematic representation of the fragments prepared in this study. (B) Raw ellipticity data (*θ*_obs_) obtained by full length EspB (circles), EspB1–176 (squares) and EspB177–312 (triangles). The protein concentration for each protein was 15 µM. Broken line indicates the sum of the spectra of EspB1–176 and EspB177–312. (C) Another representation of spectra shown in panel B expressed as mean residue ellipticity, [*θ*].

For disorder probability prediction, we used three different algorithms including PONDR [29]–[31] (http://www.pondr.com/), IUPred [32] (http://iupred.enzim.hu/) and POODLE-L [33] (http://mbs.cbrc.jp/poodle/poodle.html) (Fig. 4). Intriguingly, all three algorithms predicted that the N-terminal half of EspB has a low disorder probability, i.e. relatively high propensity to form ordered structures. By contrast, the C-terminal half of this protein was predicted to have a high propensity to form a disordered structure. To confirm the results of these predictions, we prepared two fragments of EspB corresponding to the amino acid sequences from M1 to T176 (EspB1–176) and from T177 to G312 (EspB177–312) (Fig. 5A) using an *E. coli* expression system and analyzed their ability to form secondary structures by far-UV CD (Figs. 5B and 5C).

As expected, the far-UV CD spectrum of EspB1–176 showed the minima around 210 and 222 nm which are indicative of the presence of a α-helical structure, whereas that the spectrum of EspB177–312 was typical of an unfolded protein with a minimum around 200 nm (Fig. 5B). Furthermore, the sum of the CD spectra of EspB1–176 and EspB177–312 almost completely reproduced the spectrum of full length EspB (Fig. 5B, broken line). This observation indicates that the α-helical structures in the N-terminal region are almost independent from the C-terminal unstructured region. Hence, the overall shape of the molecule comprises a partially folded N-terminal region with a significant amount of α-helical secondary structure and a largely unstructured C-terminal region. Importantly, EspB1–176 includes the region that was shown to be the α-catenin binding site [2] (from residue number 1 to 98, see also Fig. 1), suggesting that the α-helical region of this protein should be, at least partly, involved in the binding site for α-catenin.

### Structural Properties of α-Catenin Binding Site of EspB by Protein Dissection Analysis

To further refine the structural properties of the regions required for α-catenin binding in EspB, we prepared several short fragments of EspB, each 20 amino acids in length, covering the first 100 residues from the N-terminus (Fig. 5A). To facilitate the estimation of peptide concentration, all the prepared fragments were modified by FITC through the N-terminal α-amino group. This modification was also useful in the study of fluorescence anisotropy as described later.

**Figure 6:**
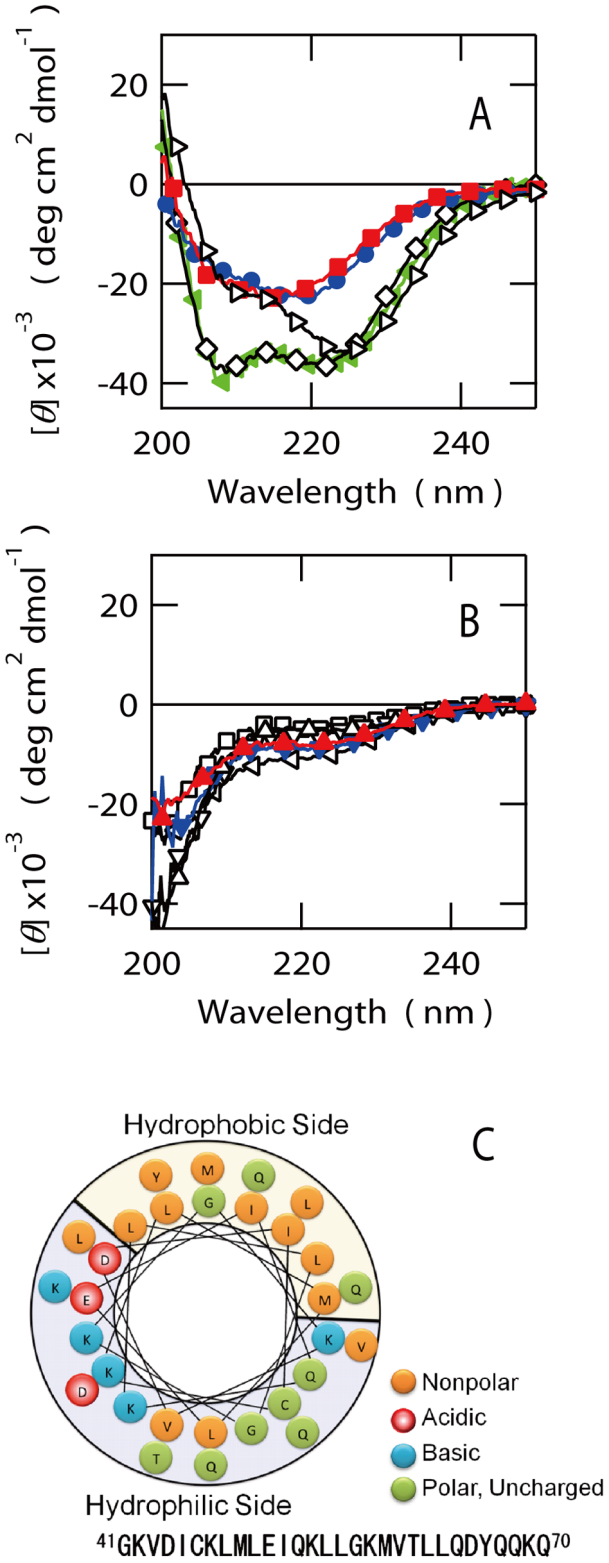
Structural properties of short fragments of EspB. (A) Far-UV CD spectra for the peptides assuming α-helical structures. EspB41–60 (l>), EspB51–70 (◊), EspB61–80 (•), EspB71–90 (▪), EspB41–70 (◂). (B) Spectra for the peptides assuming unfolded structures. EspB1–20 (□), EspB11–30 (Δ), EspB21–40 (∇), EspB31–50 (<l), EspB81–100 (▴), EspB91–110 (▾). (C) Helical wheel representation of EspB41–70, indicating the amphipathic nature of this region.

Far-UV CD spectra of the four fragments, covering the region from G41 to E90 (i.e. EspB41–60, EspBN51–70, EspBN61–80, EspBN71–90), indicated the presence of a significant amount of secondary structures (Fig. 6A). By contrast, the other fragments gave spectra corresponding to highly unfolded structures (Fig. 6B). According to the ellipticity around 222 nm, both EspB41–60 and EspB51–70 have significantly higher α-helical propensities than those of EspB61–80 and EspB71–90. Interestingly, the CD spectrum of EspB41–60 indicates intensity minima at 222 nm which is greater than that at 208 nm. The ellipticity ratio at 222 vs 208 nm ([*θ*]_222_/[*θ*]_208_) larger than 1 is possibly an indication of the formation of either the coiled-coils or other assemblies of helices [34]. Since [*θ*]_222/_[*θ*]_208_ of EspB41–60 is 1.8, the fragment may consist of such rather constraint helices. This and the fact that the SAXS for whole EspB indicates the formation of monomeric structure ruled out the possibility for the formation of oligomeric coiled-coil for EspB41–60.

Then, we calculated the secondary structure contents from the CD spectra of the EspB fragments using CDpro software package (Table 2) [17]. This analysis predicted that EspB1–20, EspB11–30, EspB21–40, EspB31–50, EspB81–100 and EspB91–110 possess relatively small amount of secondary structures and nearly 20–30% of β-strand and 10–20% of α-helix contents but with 55–60% of other structures including unordered structures and turns. EspB41–60 and EspB51–70 exhibited the highest α-helical contents of 80–90%. Although the spectra for EspB61–80 and EspB71–90 were apparently different from typical α-helical structure and rather similar to β-rich structure, CDpro suggested that the β-strand contents of these peptides are small (∼15%) and α-helical structures are still dominated (∼44%). These results indicates that the α-helical structures of EspB are probably localized around the regions from G41 to E90. For some peptides, the β-strand contents are negative values but the absolute intensities are very small. These values, therefore, can be considered as zero.

**Table 2:** Secondary structures of EspB and its peptide fragments estimated from the results of far-UV CD or amino acid sequences.

Peptide	α-Helix^a^	β-Strand^b^	Turns^c^	Unordered^d^	Helix Propensity^e^	*N*_H_ f
	(%)	(%)	(%)	(%)	(%)	
Whole EspB	22.9±1.2	22.4±1.6	21.8±0.8	34.3±1.1	4.98	76
EspB1–176	35.7±0.9	14.2±1.4	21.1±0.9	28.9±1.1	7.56	70
EspB177–312	9.1±3.6	29.9±1.2	23.5±3.0	37.8±3.7	4.72	14
EspB1–20	9.6±6.6	26.4±3.0	23.0±5.3	40.6±4.5	0.12	2
EspB11–30	11.7±6.3	27.2±6.8	22.6±2.2	39.5±0.9	0.32	2
EspB21–40	12.0±6.4	25.3±5.1	22.5±1.2	38.4±1.1	0.63	2
EspB31–50	24.9±3.3	16.5±4.5	22.5±2.6	35.8±1.8	0.56	5
EspB41–60	81.6±9.7	5.3±5.8	5.6±5.1	7.9±5.0	25.86	16
EspB51–70	89.3±7.0	−2.3±4.9	7.7±8.1	9.6±10.9	0.81	18
EspB61–80	44.0±13.1	15.1±7.7	15.7±3.6	25.7±8.7	3.10	9
EspB71–90	44.1±7.3	14.6±9.9	18.1±3.0	23.4±8.9	0.62	9
EspB81–100	19.1±1.6	22.1±2.9	21.5±2.6	37.4±1.2	5.28	4
EspB91–110	15.1±4.2	21.0±10.3	22.1±1.3	40.8±8.9	0.44	3
EspB41–70	93.9±6.0	−0.5±3.5	8.3±7.6	5.3±7.4	23.48	28

a Sum of the contents of regular and distorted α-helix estimated by CDpro [17] based on the far-UV CD spectra.

b Sum of the contents of regular and distorted β-sheet estimated by CDpro [17] based on the far-UV CD spectra.

c Contents of turns estimated by CDpro [17] based on the far-UV CD spectra.

d Contents of unordered structures estimated by CDpro [17] based on the far-UV CD spectra.

e α-Helical propensities calculated by AGADIR algorithm [35]–[39].

f Number of residues involved in α-helix calculated from the α-helix content estimated by CDpro [17] and the total length of each polypeptides. Total length of EspB, EspB1–176 and EspB177–312 are 332, 197 and 156 amino acids including additional sequences of N-terminal his-tag.

We also compared the results of CDpro analysis with the α-helical propensities of fragments predicted from the amino acid sequences using AGADIR algorithm [35]–[39]. The result of AGADIR prediction against the sequence of whole EspB was consistent with the results from the CD spectra of EspB fragments, in which the region around G41 to E90 should have high α-helical contents. The results of the AGADIR predictions against individual peptides qualitatively agree well but quantitatively less well to the estimated α-helix contents by CDpro.

**Figure 7:**
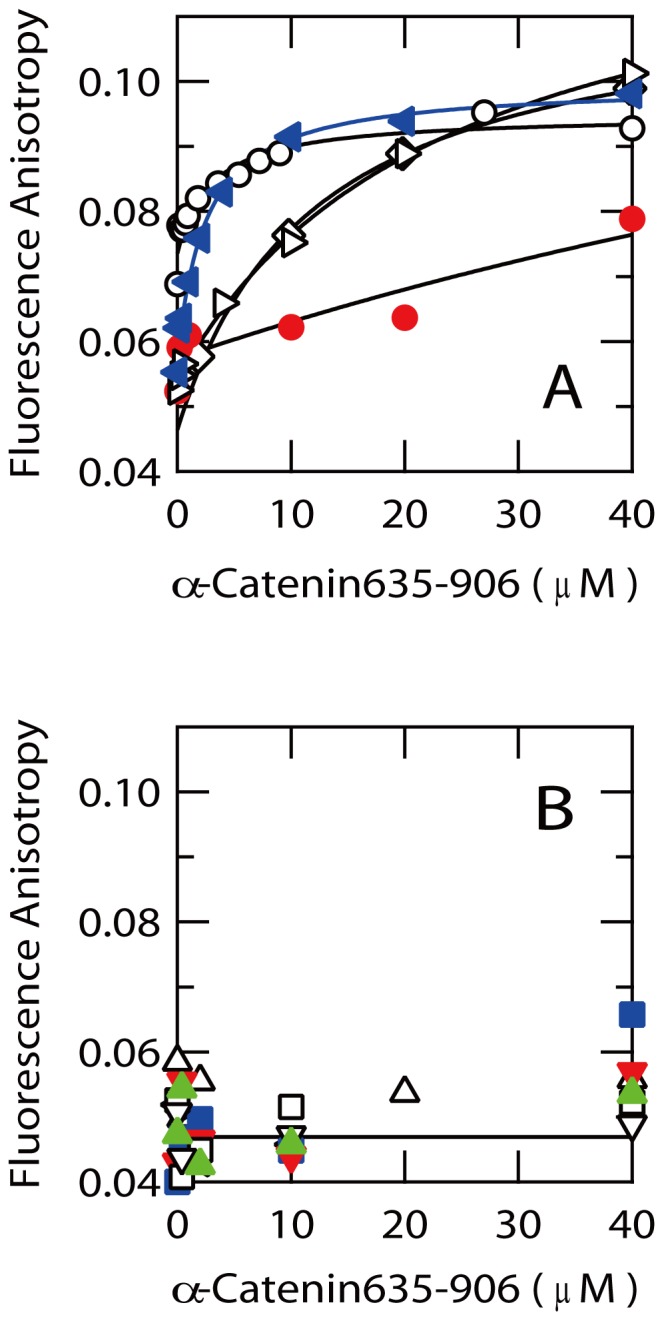
Affinity of the EspB fragments to α-catenin635–906 analyzed by fluorescence anisotropy. Each EspB peptide was modified with a fluorescein moiety at the N-terminus. (A), Titration curves for the peptides bound to α-catenin. EspB (○), EspB41–60 (l>), EspB51–70 (◊), EspB61–80 (•), EspB41–70 (◂). (B) Titration results obtained for the peptides unable to bind to α-catenin635–906. EspB1–20(□), EspB11–30 (Δ), EspB21–40 (∇), EspB31–50 (<l), EspB71–90 (▪), EspB81–100 (▴), EspB91–110 (▾).

Next, we aimed to identify the fragments that preferentially bind to the C-terminal vinculin homology domain of α-catenin (α-catenin635–906), which includes the target recognition region of intact EspB [2], using fluorescence anisotropy of the FITC-labeled EspB fragments (Fig. 7). This experiment clarified that EspB41–60, EspB51–70 and EspB61–80 bind to α-catenin635–906 (Fig. 7A) although the affinities of EspB41–60 and EspB51–70 were rather higher than that of EspB61–80. Importantly, these two fragments including EspB41–60 and EspB51–70 which indicated the high affinity to α-catenin635–906 showed the far-UV CD spectra indicative of significant α-helical propensities (Fig. 6A). However, the α-helical propensity of EspB61–80 was lower than that of EspB41–60 and EspB51–70. Interestingly, the EspB fragments which showed the CD spectra characteristic of unfolded structures (Fig. 6B) all displayed negligible affinity to α-catenin635–907 (Fig. 7B). Thus, the data showed a strong relationship between α-helical propensities of the EspB fragments and their binding abilities to α-catenin. The *K*_D_ values of EspB41–60 and EspB51–70 were 23±2 and 12±3 µM, respectively. These values are five to ten times higher than that of wild type EspB (2.9±0.3 µM), suggesting that neither of the fragments completely mimic the function of intact EspB. The affinity of EspB61–80 was too weak to obtain the reliable *K*_D_ value. An extremely high concentration of α-catenin635–906 would be required to determine an accurate *K*_D_ value for this peptide using our experimental conditions.

To further refine the regions of EspB that can reproduce the affinity of intact EspB, we prepared longer fragments corresponding to G41 to E70 (EspB41–70). The far-UV CD spectrum of EspB41–70 was consistent with the formation of α-helical structures (Fig. 6A). The analysis of CDpro indicated that the α-helical content of EspB41–70 becomes 93.9%, i.e. about 28 amino acid residues may be involved in α-helical structures in EspB41–70. AGADIR program also predicted that this fragment possess high helix propensity (Table 2). Interestingly, EspB41–70 displayed an unique thermal response similar to that of whole EspB in the presence and absence of GdnHCl (Fig. 2). The estimated Δ*C*_p_ value for EspB41–70 was 0.87±0.02 kJ mol^−1^ K^−1^. This value is rather similar to that of whole EspB (0.34±0.01 kJ mol^−1^ K^−1^). We also estimated *m* values for EspB and EspB41–70 upon unfolding reaction by GdnHCl and the Δ*G*_water_ at 20°C in the absence of GdnHCl from the linear relationship between Δ*G*_20°C_ and GdnHCl concentration, [GdnHCl], i.e. 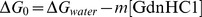 (Fig. 1D). Δ*G*_water_ of EspB (5.5±0.7 kJ mol^−1^) was smaller than that of EspB41–70 (13.0±0.2 kJ mol^−1^). Thus, the presence of additional regions at the N- and C-terminal may rather destabilize the relatively rigid α-helical structure formed around G41 to Q70. The *m* values are considered to be correlated with the change in the accessible surface area upon unfolding of a protein. Interestingly, the *m* value of EspB (1.6±0.2 kJ mol^−1^ M^−1^) was closely similar to the value of EspB41–70 (2.1±0.1 kJ mol^−1^ M^−1^), suggesting that the structural elements responsible for the rather cooperative thermal response of EspB assume the structure similar to that of EspB41–70. These results suggested that the sequence around G41 to Q70 by itself could form the core of rigid α-helical structure formed in EspB. Moreover, the *K*_D_ value of EspB41–70 for α-catenin was 2.5±0.3 µM, which is very close to the value determined for intact EspB (2.9±0.3 µM) (Fig. 7A). These results suggest that the sequence around G41 to Q70 of EspB must be the core α-helical region which is also important for the recognition of α-catenin. A close analysis of the sequence from G41 to Q70 clarified the possible formation of an amphipathic α-helix in this region (Fig. 6C) if it assumes extended α-helical structure. However, it is unclear if such an extended amphipathic α-helical structure can be formed under normal solution conditions.

**Figure 8:**
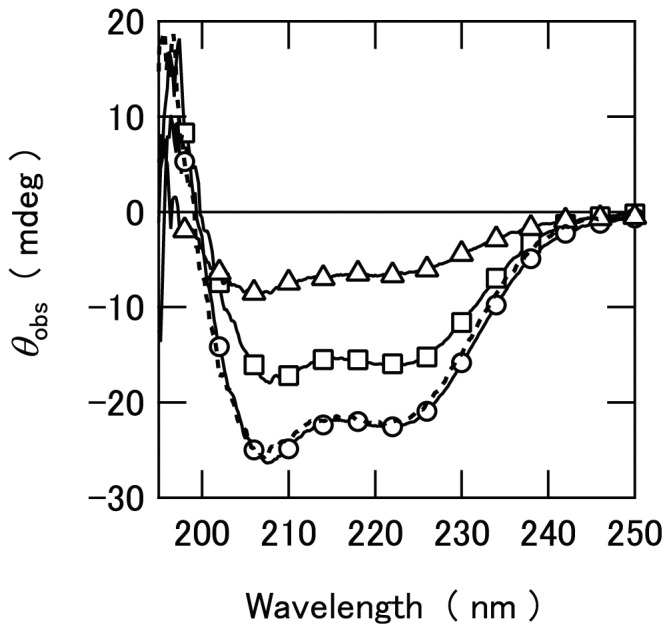
Far-UV CD spectrum of the EspB/α-catenin635–906 complex. The spectra of EspB (Δ), α-catenin635–906 (□), and a complex of EspB and α-catenin635–906 (○) are shown. Broke line indicates the calculated sum of the spectra of EspB and α-catenin635–906. The concentrations of both EspB and α-catenin635–906 were 3.2 µM.

We then analyzed if any additional secondary structures are induced for EspB upon binding to α-catenin based on far-UV CD spectra. Interestingly, the far-UV CD spectrum for the solutions containing of α-catenin635–906 and EspB in 1 to 1 ratio coincide very well with the sum of the individual spectra obtained for α-catenin635–906 and EspB (Fig. 8). This clearly indicates that no additional secondary structures are formed for these proteins upon formation of α-catenin/EspB complex.

## Discussion

In general, proteins synthesized in biological systems spontaneously folds into well-ordered structures that are unique to their amino acid sequences, thereby conferring distinctive functions. However, our data suggest that EspB possesses highly unique structural properties. Specifically, the N-terminal region around G41 to E70 of EspB has a significant amount of α-helix contents whilst the C-terminal region is largely unstructured. The analysis of EspB fragments further suggested that the α-helical region is directly involved in the binding with α-catenin.

A relatively cooperative thermal response including cold denaturation phenomena observed for EspB and its fragment of EspB41–70 suggests the presence of small but distinctively rigid structures around G41 to Q70 that is stabilized by hydrophobic interactions. However, the contribution of such hydrophobic contacts particularly among the sidechains may be very little since the values of Δ*C*_p_ were extremely small (Table 1). The length of EspB41–70 is 30 amino acid long and about 28 amino acid residues will be involved in α-helical structures according to the secondary structure prediction by CDpro. If this estimate is correct, the most parts of this fragment assumes α-helical structure and one of the extreme patterns should be an long extended α-helix. Such an extended α-helix may possess only little number of sidechain-sidechain contacts and the contribution of hydrophobic effects to the stabilization should be less high. It is of question if such an extended α-helix structure by a single chain is stabilized by the mechanism same as that of the globular protein even though the helix is sufficiently stable.

Interestingly, secondary structure prediction from the CD spectrum of EspB indicated that about 76 amino acid residues should be involved in α-helical structures within 332 amino acid sequence of EspB. Therefore, although the thermal unfolding behavior and α-catenin binding of EspB can be almost successfully explained by the contribution of the sequences from G41 to Q70, some other parts of the protein should be involved in the formation of additional α-helices. These regions may be rather less organized and behave like the partially folded intermediates of globular proteins, namely the “molten globule” states [10]–[12], [27]. Interestingly, SAXS data revealed that, although the molten globule states of globular proteins are as compact as the well-defined native states, EspB assumes an extremely extended conformation with the *R*_g_ value closely similar to the ideal random coil structures. In this sense, the less organized regions of EspB should be in the conformational states similar to the “premolten globule” states [13],[40] which are only transiently accumulated at the very early stage of folding of globular proteins. Such an extended partially folded structure of EspB should be dominantly stabilized by the local interactions formed between amino acids close to each other in the amino acid sequence rather than the nonlocal interactions formed by the residues apart from each other in the sequence.

Taken together, most parts of EspB assume an extended premolten globule-like structures with significant amount of α-helix and the region of G41 to Q70 forms relatively organized α-helical structures that are directly responsible for α-catenin binding. This result is consistent with the observations for various intrinsically disordered proteins that indicate the regions with high propensity to form secondary structures tend to be functionally important [41].

Highly unstructured intrinsically disordered proteins without having any secondary and tertiary structures often show the formation of well-defined structures upon binding to their target molecules in a manner of either “*induced fit*” or “*conformational selection*” [42]. In the former case, the unstructured protein initially binds to its target molecule as an unfolded structure followed by the formation of well-defined three dimensional structure on the target molecule. Whereas, in the latter case, the particular structure that is only transiently formed among the ensemble of the fluctuating structure of the unstructured protein is selectively recognized by the target molecule. The term, “*coupled folding and binding*” is recently used when the secondary structures (usually α-helix) are formed by induced-fit mechanism [43]–[44]. No additional secondary structure was induced neither for EspB nor α-catenin635–906 upon formation of their complex structure according to far-UV CD spectra. This result suggests that the binding of EspB to α-catenin proceeds by the recognition of the preformed secondary structure in EspB by α-catenin. This reaction may be classified into an extreme case of “*conformational selection*” or possibly called “*folding before binding*” in which the formation of α-helix structure that is similar to the helix formed in the protein complex [45]. However, the current data cannot rule out the possibility that additional conformational changes like rearrangements of sidechain orientation or helix curvature without changing total α-helical content may take place in a manner of *induced fit* reaction even in the case of EspB. This type of reaction modes that is initially driven by conformational selection followed by induced fit has been proposed for the interaction between transactivation domain of tumor suppressor p53 and the nuclear coactivator binding domain of cyclic-AMP response element binding protein from the result of molecular dynamic simulation [46]. Such a combined mechanism could be the major mechanisms of molecular recognitions particularly for intrinsically disordered proteins having partially folded regions similar to the molten globule or premolten globule states where preformed partially folded structures are involved in binding process. The structural details on the free and complex forms of EspB as well as the kinetic analysis for α-catenin binding are required to clarify the more realistic mechanism for α-catenin binding of EspB.

Interestingly, α-catenin binds to β-catenin through its N-terminal vinculin homology domain whereas EspB binds to the C-terminal vinculin homology domain. This fact suggests that the binding of EspB to the C-terminal region of α-catenin should induce the conformational change around the N-terminal region of α-catenin to promote the dissociation of α-catenin from β-catenin. The analysis of 3D structure of EspB/α-catenin complex using e.g. X-ray crystallography will clarify the mechanism of the dissociation of α-catenin from E-cadherin/β-catenin complex by the presence of EspB.

In this paper, we identified that only about 30 amino acid residues in EspB play important role for α-catenin binding which probably promotes the rearrangement cellular morphology. Then, what is the role of other regions of EspB? As mentioned earlier in this paper, EspB is a multifunctional protein which is involved in pore-formation [7], actin reorganization [2],[3],[8] and inhibition of phagocytosis [5]. The analysis demonstrated here focused on the actin reorganization through binding of α-catenin. Previous analysis on enterophathogenic *E. coli* (EPEC) indicated that the region from I159 to L218 in EPEC EspB is important for binding to myosin [5]. The regions in EHEC EspB corresponding to this region is relatively well conserved and are assigned to V158 to R109 [5] (Fig. 1). Thus, this region in EHEC EspB can also involved in inhibition of phagocytosis by binding to myosin proteins. The regions responsible for other binding partners of EspB are still unknown, but the regions other than G41–Q70 or V158 to R109 can be also required for recognition of various other proteins. Simultaneous binding of various other proteins using different binding site can also important for all the phenomena that EspB promotes. Then, why EspB has to assume less-organized extended structures with partially folded α-helices? The observations of various other effectors that are secreted through the type III secretion systems of other infectious bacteria suggest that they tend to form less-organized structures as we observed for EspB [1]. This fact implicates that the conformational disorder of T3SS effectors are required for effective secretion of these proteins through the narrow central channel of T3SS needles with inner diameters of ∼20 Å [47]. This size allows the secretion of the globular proteins with <15 amino acids in length or the hydrodynamic radius (*R*_h_) of <10 Å, according to the empirical correlation of 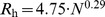 (Å) where *N* is the length of a protein [48].

In conclusion, we identified that the functionally important regions of EspB forms α-helical structures stabilized mainly by local interactions around G41 to Q70 and other regions are less well-organized. As a consequence, the overall structure of EspB is as extended as random coil structures. This result is highly relevant for understanding the mechanism of α-catenin recognition by EspB. Further analysis for the structural property of the complex between EspB and α-catenin will provide further detail of the mechanism of α-catenin binding by EspB. Such information will be also critical for understanding more precise structural details of unbound form of EspB in solution.
